# Clinical significance of extranodal extension in sentinel lymph node positive breast cancer

**DOI:** 10.1038/s41598-020-71594-7

**Published:** 2020-09-07

**Authors:** Xia Yang, XiaoXi Ma, Wentao Yang, Ruohong Shui

**Affiliations:** 1 Department of Pathology, Fudan University Shanghai Cancer Center, Shanghai, China; 2 Department of Oncology, Shanghai Medical College, Fudan University, Shanghai, China

**Keywords:** Breast cancer, Cancer, Medical research, Oncology, Pathogenesis

## Abstract

The precise stage of lymph node (LN) metastasis is a strong prognostic factor in breast cancers, and sentinel lymph node (SLN) is the first station of nodal metastasis. A number of patients have extranodal extension (ENE) in SLN, whereas the clinical values of ENE in SLN in breast cancers are still in exploration. The aim of our study was to evaluate the predictive and prognostic values of ENE in SLN in breast cancers, and to investigate the feasibility of ENE to predict non-SLN metastasis, nodal burden, disease free survival (DFS) and overall survival (OS) in clinical practice. 266 cases of primary invasive breast cancer (cT1-2N0 breast cancer) underwent SLN biopsy and axillary lymph node dissection (ALND) between 2008 and 2015 were extracted from the pathology database of Fudan University Shanghai Cancer Center. ENE in SLN was defined as extension of neoplastic cells through the lymph-nodal capsule into the peri-nodal adipose tissue, and was classified as no larger than 2 mm and larger than 2 mm group. The associations between ENE and clinicopathological features, non-SLN metastasis, nodal burden, DFS, and OS were analyzed. In the 266 patients with involved SLN, 100(37.6%) were positive for ENE in SLN. 67 (25.2%) cases had ENE no larger than 2 mm in diameter, and 33(12.4%) had ENE larger than 2 mm. Among the clinicopathological characteristics, the presence of ENE in SLN was associated with higher pT and pN stages, PR status, lympho-vascular invasion. Logistic regression analysis indicated that patients with ENE in SLN had higher rate of non-SLN metastasis (OR4.80, 95% CI 2.47–9.34, *P* < 0.001). Meanwhile, in patients with SLN micrometastasis or 1–2 SLNs involvement, ENE positive patients had higher rate of non-SLN metastasis, comparing with ENE negative patients (*P* < 0.001, *P* = 0.004 respectively). The presence of ENE in SLN was correlated with nodal burden, including the pattern and number of involved SLN (*P* < 0.001, *P* < 0.001 respectively), the number of involved non-SLN and total positive LNs (*P* < 0.001, *P* < 0.001 respectively). Patients with ENE had significantly higher frequency of pN2 disease (*P* < 0.001). For the disease recurrence and survival status, Cox regression analysis showed that patients with ENE in SLN had significantly reduced DFS (HR 3.05, 95%CI 1.13–10.48, *P* = 0.008) and OS (HR 3.34, 95%CI 0.74–14.52, *P* = 0.092) in multivariate analysis. Kaplan–Meier curves and log-rank test showed that patients with ENE in SLN had lower DFS and OS (for DFS: *P* < 0.001; and for OS: *P* < 0.001 respectively). Whereas no significant difference was found in nodal burden between ENE ≤ 2 mm and > 2 mm groups, except the number of SLN metastasis was higher in patients with ENE > 2 mm. Cox regression analysis, Kaplan–Meier curves and log-rank test indicated that the size of ENE was not an independent factor of DFS and OS. Our study indicated that ENE in SLN was a predictor for non-SLN metastasis, nodal burden and prognosis in breast cancers. Patients with ENE in SLN had a higher rate of non-SLN metastasis, higher frequency of pN2 disease, and poorer prognosis. Patients with ENE in SLN may benefit from additional ALND, even in SLN micrometastasis or 1–2 SLNs involvement patients. The presence of ENE in SLN should be evaluated in clinical practice. Size of ENE which was classified by a 2 mm cutoff value had no significant predictive and prognostic values in this study. The cutoff values of ENE in SLN need further investigation.

## Introduction

Sentinel lymph node (SLN) is the first station of nodal metastasis^1,2^. Axillary SLN biopsy could accurately predict axillary lymph node status and has been established as standard treatment in patients with clinically negative lymph nodes (cN0) breast cancers^3–5^. In 2010, the results of American College of Surgeons Oncology Group (ACOSOG) Z0011 trial indicated that patients with limited disease burden on SLN (even 1–2 macrometastasis) without axillary lymph node dissection (ALND) could obtain excellent regional control, and SLN biopsy may be reasonable management for selected patients with early-stage (clinical T1N0 or T2N0) breast cancer treated with breast conserving surgery, radiotherapy and adjuvant systemic therapy^6^. Based on these results, American Society of Clinical Oncology Clinical Practice Guideline and National Comprehensive Cancer Network (NCCN) recommend that women who meet the Z0011 criteria may not undergo ALND^7,8^. Increasing number of patients undergo SLN biopsy which could avoid the underlying morbidity of ALND^9–11^.However, a subset of patients with limited disease burden on SLN may have relatively high aggressive behavior and poor survival after SLNB. Thus, it is important to explore the poor prognostic factors in breast cancers with SLNs involvement.

Extranodal extension (ENE), defined as extension of neoplastic cells through the lymphnodal capsule into the peri-nodal adipose tissue, has emerged as an important prognostic factor in several types of malignancies^12–19^. Several studies suggested that the presence and extent of ENE in SLN were significantly correlated with non-SLN metastasis and the number of involved lymph nodes in breast cancers^20–25^. However, few studies have focused on the prognosis value of ENE in SLN. The clinical significance of ENE in breast cancers is still in exploration.

The aim of this study was to establish the pathological assessment of ENE and to evaluate the clinical significance of ENE in SLN in primary invasive breast cancers, including its association with non-SLN metastasis, nodal burden, disease free survival (DFS) and overall survival (OS).

## Patients and methods

### Patients

266 consecutive patients with primary invasive breast cancers (cT1-2N0) who underwent SLN biopsy and ALND at Fudan University Shanghai Cancer Center from 2008 to 2015 were analyzed. Patients were diagnosed as clinical N0 if lymph nodes were negative by palpation, ultrasound detection and fine needle aspiration. 266 patients enrolled in this study were all cN0 and with positive SLN and additional ALND. Patients with incomplete clinical information, recurrence /metastasis at diagnosis, previous axillary surgery, or received neoadjuvant chemotherapy were excluded. Informed consent was obtained from all patients. The SLN was identified using 1% isosulfan blue dye and 99mTc-labeled sulfur colloid. SLN biopsy was performed as lymph nodes that demonstrated blue dye uptake, radiotracer uptake, or both. Imprint cytology of SLN was performed during operation. Each SLN was serially cut to tissue blocks along the short axis at 2 mm intervals and imprint was performed on both sides of each tissue block. Those patients with metastatic tumor cells on imprint slice received additional ALND. Final diagnosis of SLN was performed on paraffin-embedded tissues. All tissue blocks of SLN were fixed in formalin, embedded in paraffin, and examined using hematoxylin and eosin (H&E) staining with serial section. The pattern of metastasis (ITC, micrometastasis or macrometastasis) was analyzed. If SLN macrometastasis was recognized on paraffin-embedded slices in patients with negative results of imprint cytology, these patients received additional ALND. Non-SLNs were evaluated using H&E staining.

All patients were treated with surgery (modified radical mastectomy with SLN and ALND), with or without radiotherapy, systematic chemotherapy, endocrine therapy, and targeted therapy according to National Comprehensive Cancer Network (NCCN) guideline recommendations. 50.4% (134/266) patients underwent radiotherapy, 80.8% (215/266) patients underwent chemotherapy. 78.2% (208/228) patients underwent endocrine therapy, and 16.5% (44/266) patients underwent anti-HER2 targeted therapy. Treatment details were displayed in Table 1.

**Table 1:** Correlations between ENE in SLN and clinicopathological characteristics.

Variables	No. of patients (%)	Extranodal extension		*P* value
		Negative	Positive	
Total population	266 (100)	166 (62.4%)	100 (37.6%)	
Median age (Y)	49 (27–83)	50 (27–83)	49 (27–77)	0.282
**Pathological T stage**				
T1	132 (49.6)	94 (35.3%)	38 (14.3%)	0.003
T2	134 (50.4)	72 (27.1%)	62 (23.3%)	
**Histological grade**				
2	159 (59.8)	101 (38.0%)	58 (21.8%)	0.647
3	107 (40.2)	65 (24.4%)	42 (15.8%)	
**N stage**				
1	186 (69.9)	145 (54.5)	41 (15.4%)	< 0.001
2	66 (24.8)	23 (8.6%)	43 (16.2%)	
3	14 (5.3)	2 (0.7%)	12 (4.6%)	
**Molecular subtype**				
Luminal A-like	83 (31.2)	55 (20.7%)	28 (10.5%)	0.401
Luminal B-like	139 (52.2)	79 (29.7%)	60 (22.5%)	
HER2 overexpression	22 (8.3)	17 (6.4%)	5 (1.9%)	
TNBC	22 (8.3)	15 (5.6%)	7 (2.7%)	
**ER status**				
Negative	52 (19.5)	38 (14.3%)	14 (5.2%)	0.077
Positive	214 (80.5)	128 (48.1%)	86 (32.4%)	
**PR status**				
Negative	72 (27.1)	55 (20.7%)	17 (6.4%)	0.004
Positive	194 (72.9)	111 (41.7%)	83 (31.2%)	
**HER2 status**				
Negative	215 (80.8)	133 (50.0%)	82 30.8%)	0.706
Positive	51 (19.2)	33 (12.4%)	18 (6.8%)	
**Lympho-vascular invasion**				
Negative	139 (52.3)	108 40.6%)	31 (11.7%)	< 0.001
Positive	127 (47.7)	58 (21.8%)	69 (25.9%)	

*ER* estrogen receptor, *PR* progesterone receptor, *HER2* human epidermal growth factor receptor 2, *ENE* extranodal extension, *SLN* sentinel lymph node, *TNBC* triple negative breast cancer.

### Patient characteristics

The clinicopathological variables included age, tumor size, histological grade, estrogen receptor (ER), progesterone receptor (PR), human epidermal growth factor receptor 2 (HER2) status, lympho-vascular invasion, the number and pattern (ITC, micrometastasis or macrometastasis) of SLN metastases, and the presence and size of ENE in involved SLN. ER and PR were judged as positive if ≥ 1% of tumor cells showed nuclear staining^26^. HER2-positive status was defined as 3 + score by IHC or HER2 gene amplification by fluorescent in situ hybridization (FISH)^27^. According to the expression of ER, PR, HER2, Ki67, the patients were classified as luminal A-like, luminal B-like, HER2 overexpression and triple negative molecular subtypes.

**Figure 1:**
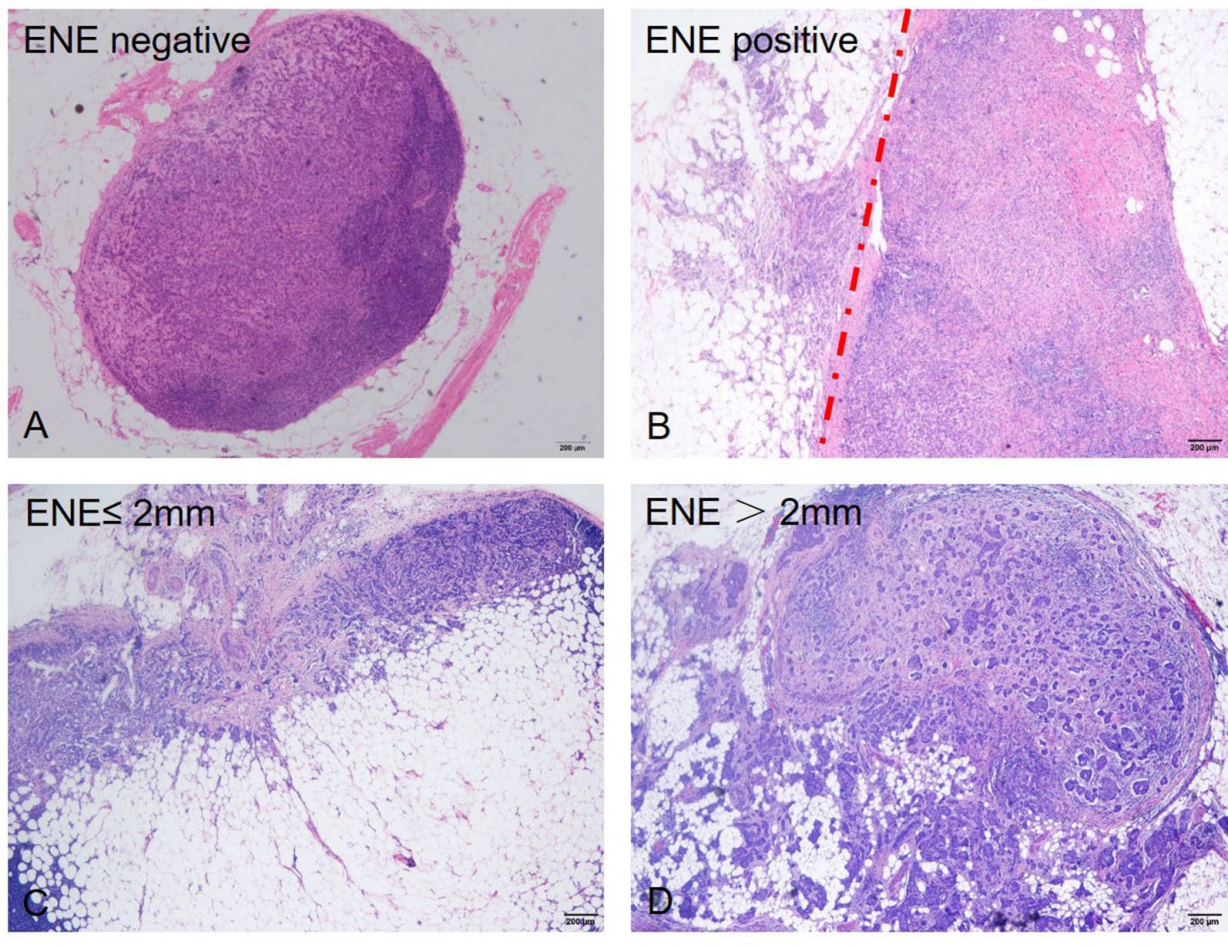
(**A**) Involved sentinel lymph node (SLN) without ENE, (**B**) SLN with ENE, (**C**) SLN with ENE ≤ 2 mm, (D) SLN with ENE > 2 mm. Original magnification: 200x.

Clinicopathological variables were reviewed by two certified experienced breast pathologists (Wentao Yang and Ruohong Shui). The number and pattern of SLN metastasis, the presence of ENE in involved SLN were reviewed by two certified breast pathologists (Xia Yang and XiaoXi Ma) in a blind manner. The pattern of SLN involvement was according to the maximum size of involved SLNs. ITC was defined as tumor cell deposits no larger than 0.2 mm in diameter or less than 200 tumor cells in the slice, micrometastasis was defined as metastatic lesions larger than 0.2 mm and no larger than 2.0 mm in diameter or more than 200 tumor cells in the slice, and macrometastasis was defined as metastatic lesions larger than 2 mm in diameter. Extranodal extension was defined as positive if metastatic tumor invasion of extranodal fat with or without associated desmoplastic stromal response (ie, inflamed granulation tissue and/or fibrosis). The size of ENE was measured as the highest or widest diameter of the invasive front of ENE and categorized as no larger than 2 mm and larger than 2 mm groups (Fig. 1).

### Study end points

This study aimed to evaluate the association between ENE in involved SLN and non-SLN metastases, nodal burden, DFS and OS. After surgery for primary breast cancer, patients were assessed for disease recurrence or/and metastasis in accordance with standard clinical practice. DFS was defined as the time from surgery to events including local recurrence which had ipsilateral breast tumor recurrence or distant recurrence, or death resulting from any cause (whichever occurred first). OS was defined as the time from surgery to death from any cause.

### Statistical analysis

Categorical variables were analyzed using X^2^ test or Fisher exact test. Quantitative variables were evaluated using t test, and continuous variables were compared in different ENE groups using t test. Logistic regression analysis was used to evaluate relationships between ENE and non-SLN involvement in a multivariate model. Durations of DFS and OS were analyzed using the Kaplan–Meier method. Differences in DFS and OS were assessed using the log-rank test. Cox regression analysis was used to evaluate relationships between ENE in SLN and prognosis in a multivariate model. All statistical tests were two-sided and the statistical significance was defined as *P* < 0.05. All statistical analyses were carried out using SPSS statistical software (version 20.0; SPSS INC., Chicago, IL). All figures were depicted using Graphpad Prism (GraphPad Software).

## Results

### Extranodal extension in SLN and clinicopathological features

The clinicopathological characteristics of 266 primary invasive carcinomas were listed in Table 1. All patients were female. The median age was 50 years, ranging from 27 to 83 years. 100/266(37.6%) cases were positive for ENE in SLN. 67 (25.2%) cases had ENE ≤ 2 mm in diameter, and 33 (12.4%) had ENE > 2 mm. 49.6% of patients (132/266) had a limited tumor size (no more than 2 cm), and 80.1% (213/266) of the patients had one or two positive SLN. The majority of cases (87.2%,232/266) had macrometastass in SLN. Among the clinicopathological characteristics examined in this cohort, ENE in SLN was associated with higher T and N stage, PR status, lympho-vascular invasion, comparing with the patients without ENE in SLN (Table 1). The intraclass correlation coefficient (ICC) analysis showed that the interobserver agreement of ENE assessment between two observers was excellent (ENE: ICC 0.95, 95% CI 0.88–0.98, *P* < 0.001).

### Extranodal extension in SLN and non-SLN metastasis

In the 100 patients with ENE in SLN, 77/100 (77.0%) had additional non-SLN metastasis, compared with 50/166 (30.1%) patients without ENE in SLN (*P* < 0.001). Univariate analysis indicated that ENE in SLN, pattern of SLN metastasis, numbers of involved SLN, lympho-vascular invasion, ER/PR/HER2 status were significantly associated with the presence of non-SLN metastasis. Multivariate analysis including predictive factors indicated that ENE in SLN was an independent predictor of non-SLN metastasis (OR4.80, 95% CI 2.47–9.34, *P* < 0.001). Additionally, lympho-vascular invasion and HER2 status also were significantly associated with non-SLN metastasis (*P* < 0.001, *P* = 0.010 respectively) in multivariate analysis (Table 2). In 34 patients with SLN micrometastasis cases with ENE in SLN had higher rate (1/2, 50.0%) of non-SLN metastasis, compared with ENE negative patients (5/32, 15.6%) (*P* < 0.001). In patients with 1–2 SLNs involvement, cases with ENE in SLN had higher rate (50/66, 75.8%) of non-SLN metastasis, compared with ENE negative patients (41/147, 27.9%) (*P* = 0.004). According to these findings, ENE in SLN may be used as an indicator for non-SLN metastasis in early stage breast cancers and such patients may benefit from further ALND.

**Table 2:** Correlations between ENE in SLN and non-SLN metastasis.

Variables	Univariate analysis			Multivariate analysis		
	OR	95% CI	*P* value	OR	95% CI	*P* value
**Age (Y)**						
< 50	1	–	–			
≥ 50	0.8	0.49–1.30	0.359			
**Pathological T stage**						
T1	1	–	–			
T2	1.13	0.70–1.83	0.62			
**Histological grade**						
2	1	–	–			
3	0.88	0.54–1.43	0.602			
**No. of SLN metastasis**						
≤ 2	1	–	–	1	–	–
> 2	2.84	1.50–5.37	0.001	1.59	0.73–3.44	0.243
**Pattern of SLN metastasis**						
Micrometastasis	1	–	–	1	–	–
Macrometastasis	5.09	2.03–12.75	0.001	2.1	0.69–6.39	0.193
**ENE**						
Negative	1	–	–	1	–	–
Positive	7.77	4.39–13.76	< 0.001	4.8	2.47–9.34	< 0.001
**ER status**						
Negative	1	–	–			
Positive	2.43	1.27–4.63	0.007	1.11	0.37–3.35	0.856
**PR status**						
Negative	1	–	–	1	–	–
Positive	2.93	1.64–5.23	< 0.001	1.6	0.61–4.21	0.338
**HER2 status**						
Negative	1	–	–			
Positive	0.38	0.20–0.74	0.004	0.31	0.12–0.75	0.010
**Molecular subtype**						
Luminal A-like	1	–	–			
Luminal B-like	0.78	0.54–1.60	0.78			
HER2 overexpreesion	0.26	0.09–1.37	0.053			
TNBC	0.41	0.15–1.12	0.082			
**Lympho-vascular invasion**						
No	1	–	–	1	–	–
Yes	7.23	4.21–12.43	< 0.001	6.02	3.24–11.18	< 0.001

*ER* estrogen receptor, *PR* progesterone receptor, *HER2* human epidermal growth factor receptor 2, *ENE* extranodal extension, *SLN* sentinel lymph node, *TNBC* triple negative breast cancer.

**Figure 2:**
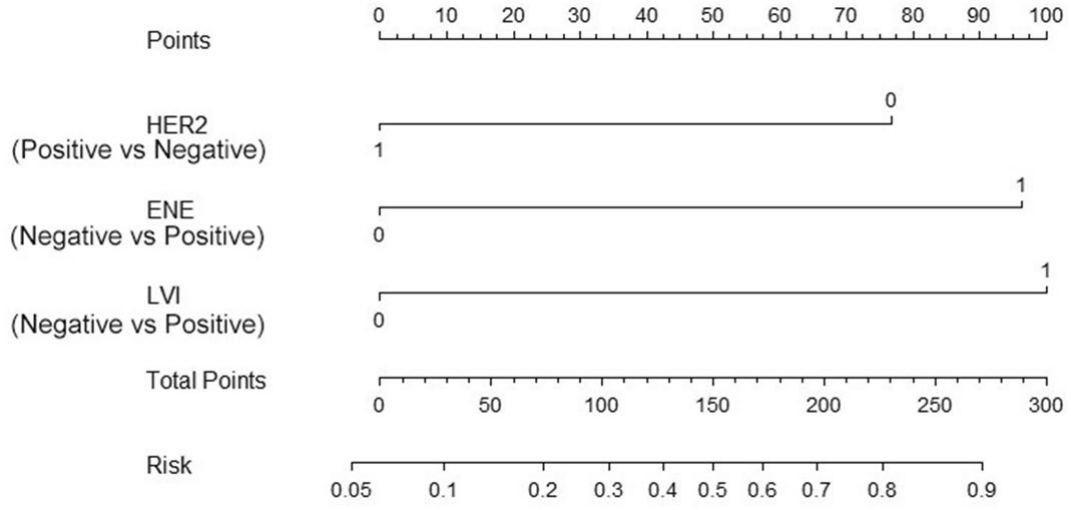
Nomogram for the prediction of non-SLN metastasis. *LVI* lymph-vascular invasion, *ENE* extra-nodal extension, *HER2* human epidermal growth factor receptor 2.

In order to build a nomogram for predicting the risk of non-SLN metastasis, 3 risk factors (ENE, HER2, lympho-vascular invasion) with statistical significance in multivariable analysis were combined. A line (line 1) was drawn upward for each risk factor (line 2–4) to acquire point values. Then, the sum of these 3 points was plotted out of the total number of points on axis 5, and a line downwards toward the risk axis (axis 6) was drawn to determine the likelihood of non-SLN metastasis for an individual patient (Fig. 2). The C-indices of the non-SLN metastasis nomogram were 0.78 (95% CI 0.71 to 0.89).

### Relationships between extranodal extension in SLN and nodal burden

Patients with ENE in SLN had higher nodal burden. ENE in SLN was associated with more involved SLN (*P* < 0.001), macrometastasis in SLN (*P* < 0.001), more non-SLN metastasis (*P* < 0.001) and more total positive LNs (*P* < 0.001), comparing with ENE negative group. Higher rate of pN2 stage was shown in ENE positive group comparing with ENE negative group (*P* < 0.001). The size of ENE subdivided by a 2 mm cutoff value had no significant correlation with nodal burden, excepting that the number of SLN metastasis (*P* = 0.032) was higher in ENE > 2 mm group. There was no significant difference in the pattern of involved SLN (*P* = 0.316), the number of non-SLN metastasis (*P* = 0.378), the number of total positive LNs (*P* = 0.057), and the rate of pN2 stage (*P* = 0.532) between ENE ≤ 2 mm and ENE > 2 mm two groups (Table 3).

**Table 3:** Correlations between ENE in SLN and Nodal burden.

Nodal burden	Extranodal extension			*P* value	
	Negative (166)	Positive (100)		Negative vs Positive	≤ 2 mm vs > 2 mm
		≤ 2 mm (67)	> 2 mm (33)		
**Pattern of SLN metastasis**					
Micrometastasis	32 (19.3%)	2 (3.0%)	0 (0.0%)	0.001	0.316
Macrometastasis	134 (80.7%)	65 (97.0%)	33 (100%)		
**No. of SLN metastasis**					
1–2	147 (88.6%)	49 (73.1%)	17 (51.5%)	< 0.001	0.032
≥ 3	19 (11.4%)	18 (26.9%)	16 (48.5%)		
**NSLN metastasis**					
Mean (SD)	0.96 (0.90)	2.66 (3.48)	4.58 (5.07)	< 0.001	0.378
**NO. (%)**					
0	116 (69.9%)	15 (22.4%)	8 (24.2%)	< 0.001	0.603
1	24 (14.5%)	13 (19.4%)	3 (9.1%)		
2	12 (7.2%)	9 (13.4%)	3 (9.1%)		
3	8 (4.8%)	6 (9.0%)	3 (9.1%)		
≥ 4	6 (3.6%)	24 (35.8%)	16 (48.5%)		
**Total positive LNs**					
Mean (SD)	2.56 (2.40)	4.87 (3.95)	7.33 (5.97)	< 0.001	0.057
**No. (%)**					
1–3	145 (87.3%)	25 (37.3%)	10 (30.3%)		
≥ 4	21 (12.7%)	42 (62.7%)	23 (69.7%)	< 0.001	0.532

*SD* standard deviation, *ENE* extranodal extension, *SLN* sentinel lymph node, *NSLN* non-sentinel lymph node.

### Extranodal extension in SLN and long-term survival

**Figure 3:**
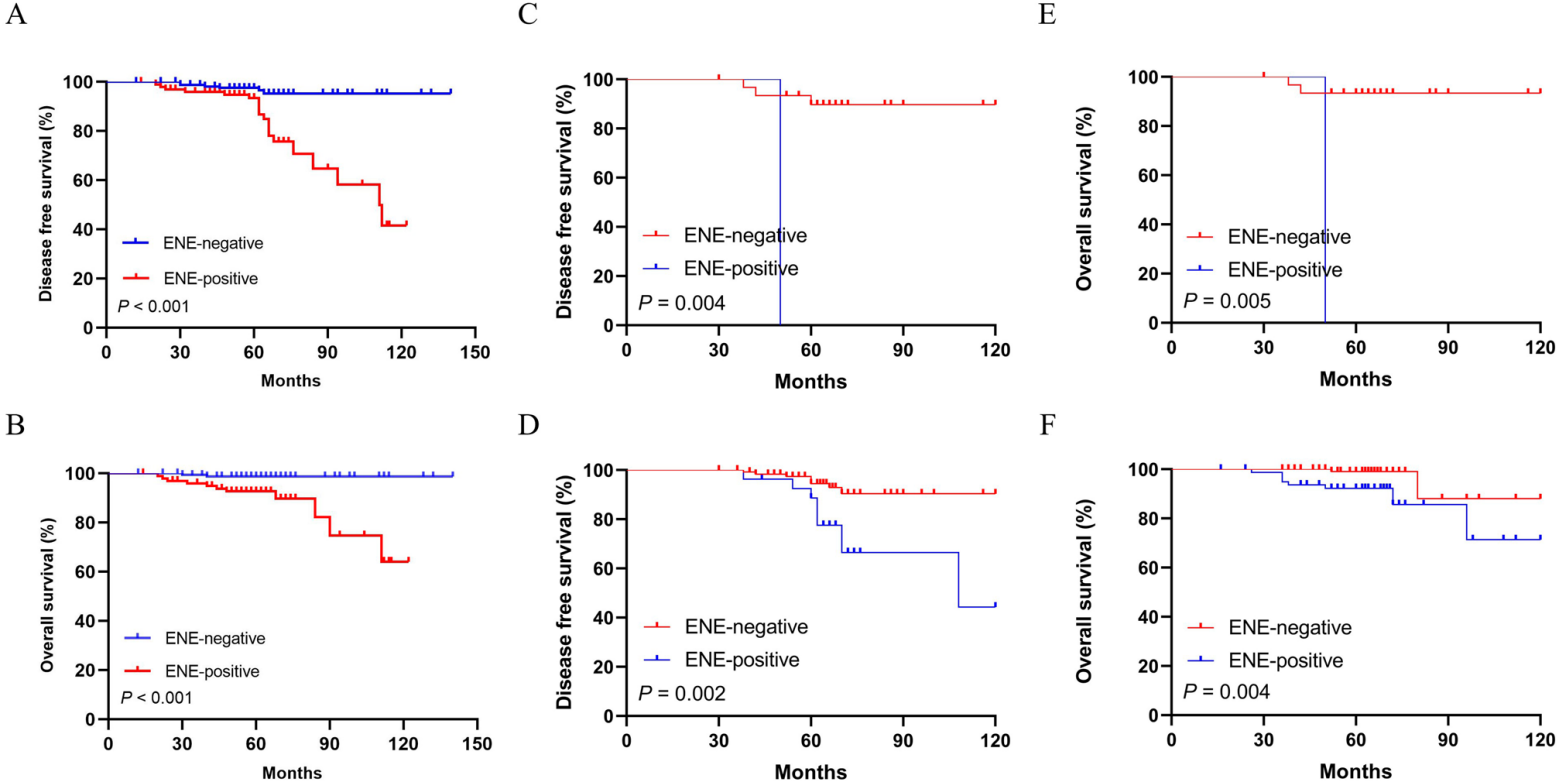
Kaplan–Meier curves depicting associations of ENE in SLN with DFS and OS in whole patients. Log-rank *P* values were shown. Comparison of survival rate for DFS (ENE negative group vs. ENE positive group: *P* < 0.001) (**A**) and OS (ENE negative group versus ENE positive group: *P* < 0.001) (**B**). Comparison of survival rate for DFS and OS (ENE negative group vs. ENE positive group: *P* = 0.004; *P* = 0.005 respectively) in patients with SLN micrometastasis (**C**,**E**) and in patients with SLN macrometastasis (ENE negative group vs. ENE positive group: *P* = *0.002; P* = 0.004 respectively*)* (**D**,**F**).

**Figure 4:**
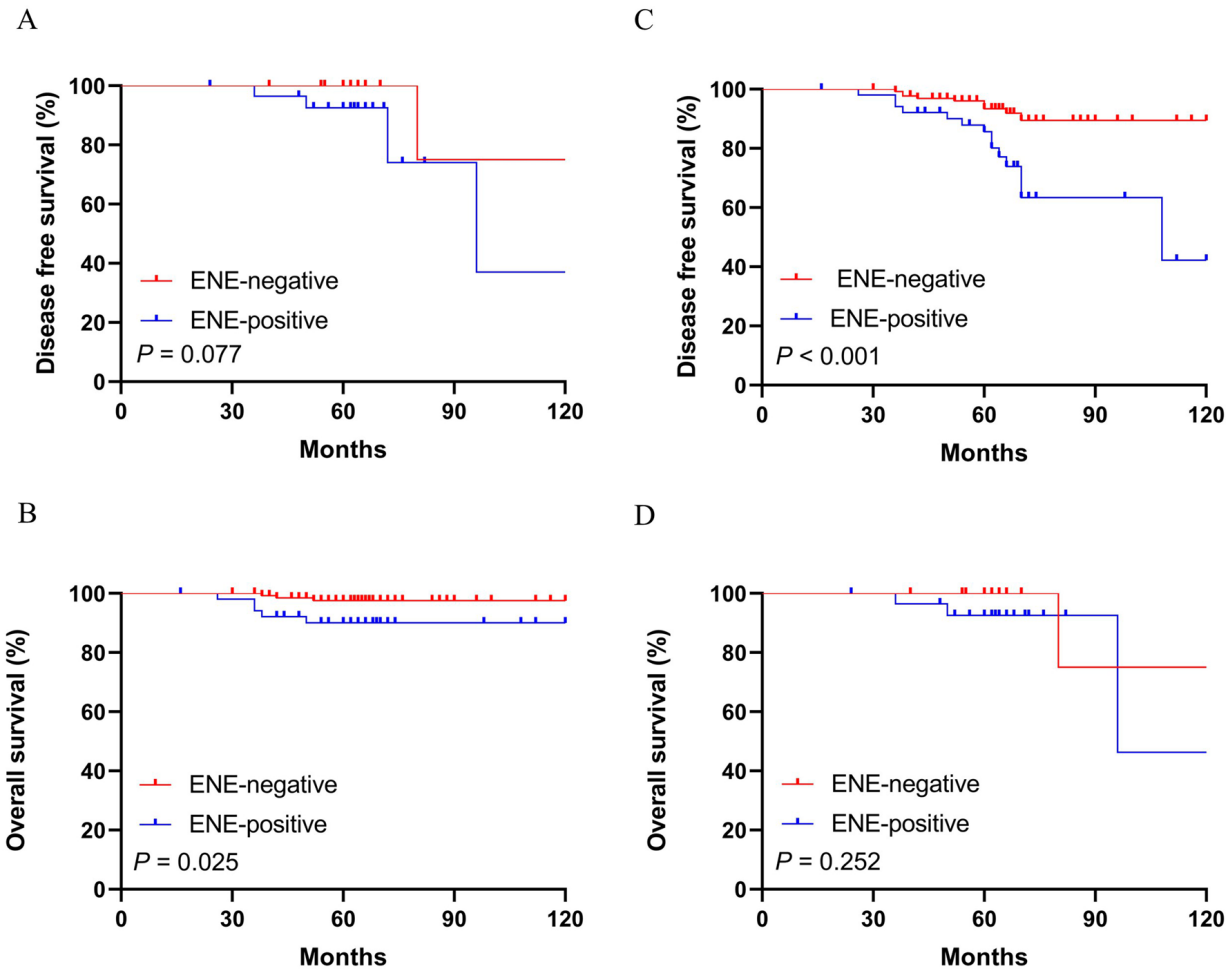
Kaplan–Meier curves depicting associations of ENE in SLN with DFS and OS in patients with different Nodal status. Comparison of survival rate for DFS and OS (ENE negative group vs. ENE positive group: *P* = 0.077; *P* = 0.025 respectively) in patients with No. of SLN metastasis ≤ 2 (**A**,**B**) and in patients with No. of SLN metastasis > 2 (ENE negative group vs. ENE positive group: *P* < 0.001; *P* = 0.252 respectively) (**C**,**D**).

**Figure 5:**
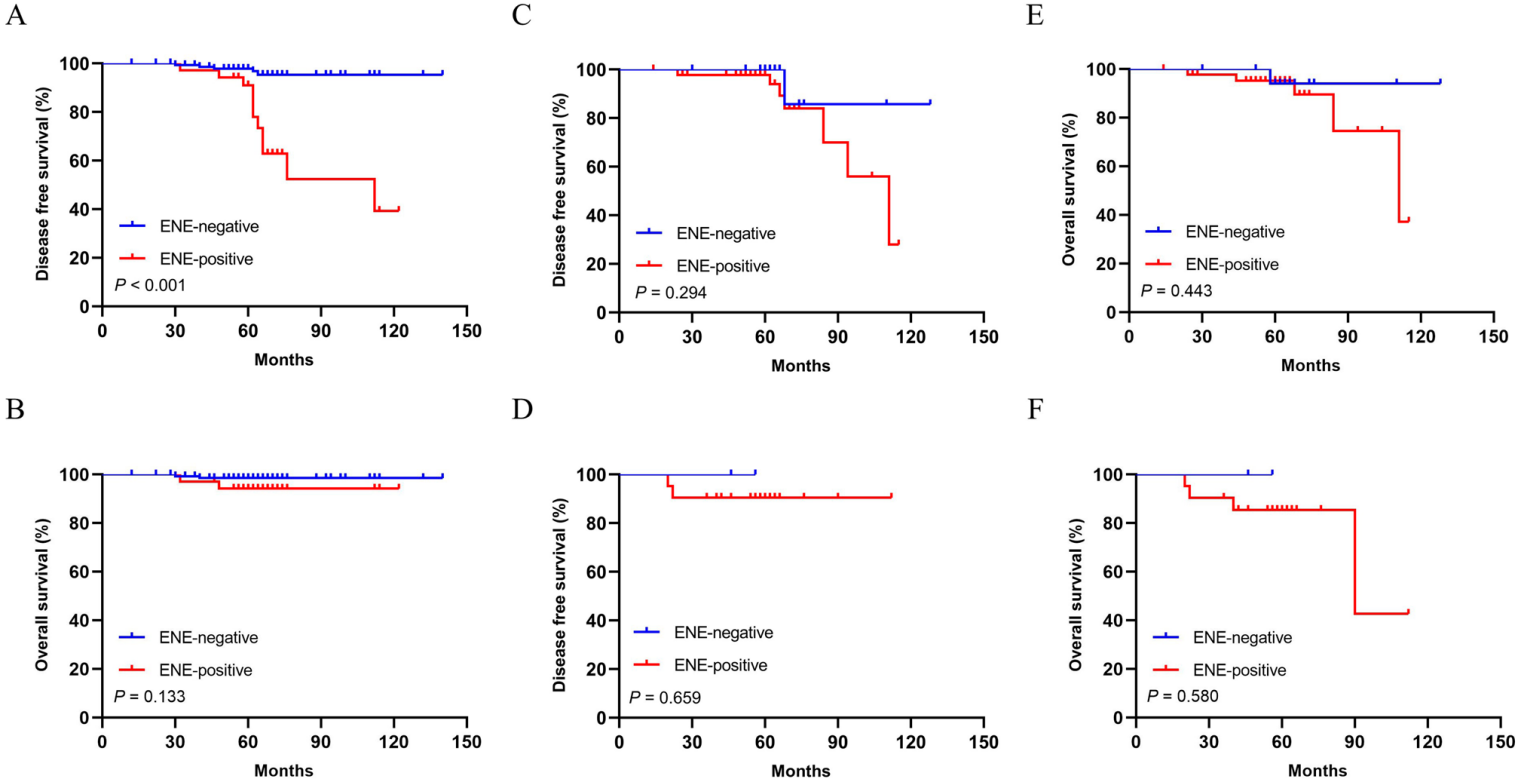
Kaplan–Meier curves depicting associations of ENE in SLN with DFS and OS in SLN positive patients with different nodal (N) stage. Comparison of survival rate for DFS (ENE negative group vs. ENE positive group: *P* < 0.001) (**A**) and OS (ENE negative group vs. ENE positive group: *P* = 0.133) (**B**) between different ENE groups in patients with pN1 stage. Comparison of survival rate for DFS (ENE negative group vs. ENE positive group: *P* = 0.294) (**C**) and OS (ENE negative group vs. ENE positive group: *P* = 0.443) (E) between different ENE groups in patients with pN2 stage. Comparison of survival rate for DFS (ENE negative group vs. ENE positive group: *P* = 0.659) (**D**) and OS (ENE negative group vs. ENE positive group: *P* = 0.580) (F) between different ENE groups in patients with pN3 stage.

Survival data were available for all patients in this cohort. Over a median follow-up of 65 months (range 8–136), 26 patients (9.8%) had local and/or distant recurrence, and 13 patients (4.9%) died during this follow-up period. Kaplan–Meier curves and log-rank test showed that patients with ENE in SLN had lower DFS and OS comparing with ENE negative group (for DFS: *P* < 0.001; and for OS: *P* < 0.001 respectively) (Fig. 3A-B). In SLN micrometastasis and macrometastasis groups, patients with ENE in SLN both had lower DFS and OS comparing with ENE negative group (for DFS: *P* = 0.004, *P* = 0.002; and for OS: *P* = 0.005, *P* = 0.004 respectively) (Fig. 3C–F). In SLN involvement ≤ 2 group, patients with ENE in SLN had lower OS and similar DFS comparing with ENE negative group (for DFS: *P* = 0.077; and for OS: *P* = 0.025 respectively) (Fig. 4A-B). In SLN involvement > 2 group, patients with ENE in SLN had lower DFS and similar OS comparing with ENE negative group (for DFS: *P* < 0.001; and for OS: *P* = 0.252 respectively) (Fig. 4C-D). In pN1 stage patients, patients with ENE in SLN had lower DFS comparing with ENE negative group, while no similar influence was observed on OS (for DFS: *P* < 0.001; and for OS: *P* = 0.133 respectively) (Fig. 5A-B). However, ENE had no significantly influence on DFS (pN2: *P* = 0.294; pN3: *P* = 0.659 respectively) (Fig. 5C-D) and OS (pN2: *P* = 0.443; pN3: *P* = 0.580 respectively) (Fig. 5E-F) in pN2 and pN3 stage patients.

Cox proportional hazards regression analyses were performed to evaluate the prognostic value of ENE in SLN in breast cancer (Table 4). It showed that ENE in SLN was significantly associated with DFS (HR5.58, 95% CI 2.24–13.90, *P* < 0.001), and OS (HR 5.08, 95% CI 2.01–20.98, *P* = 0.004) in univariate analysis. Multivariate analysis including prognostic variables confirmed that ENE in SLN was an independent predictor of DFS (HR 3.05, 95%CI: 1.13–10.48, *P* = 0.008), while no significant result was shown on OS (HR 3.34, 95%CI: 0.74–14.52, *P* = 0.092). Additionally, pT stage, histological grade, radiation therapy and endocrine therapy also were significantly associated with DFS both in univariable and multivariable analysis. Histological grade and radiation therapy were independent predictors for OS.

**Table 4:** Correlations between ENE in SLN and prognosis (DFS and OS).

Variables	Disease free survival				Overall survival			
	Univariate analysis		Multivariate analysis		Univariate analysis		Multivariate analysis	
	HR (95% CI)	*P* value	HR (95% CI)	*P* value	HR (95% CI)	*P* value	HR (95% CI)	*P* value
**Age (Y)**								
< 50	–	–			–	–		
≥ 50	0.60 (0.27–1.35)	0.216			0.31 (0.09–1.13)	0.076		
**Tumor size**								
T1	–	–			–	–		
T2	4.13 (1.56–10.99)	0.004	2.75 (1.01–7.52)	0.049	5.40 (1.48–19.62)	0.011	1.05 (0.18–6.25)	0.559
**Histological grade**								
2	–	–			–	–		
3	2.53 (1.15–5.58)	0.022	2.35 (1.10–5.45)	0.048	5.19 (1.15–23.47)	0.033	4.66 (1.68–13.17)	0.045
**N stage**								
1	–	–			–	–		
2	1.45 (0.62–3.40)	0.391			3.64 (0.98–13.58)	0.055	3.43 (0.74–11.72)	0.109
3	1.39 (0.32–6.07)	0.66			9.56 (2.38–38.35)	0.001	3.92 (0.79–14.70)	0.136
**ENE**								
Negative	–	–			–	–		
Positive	5.58 (2.24–13.90)	< 0.001	3.05 (1.13–10.48)	0.008	5.08 (2.01–20.98)	0.004	3.34 (0.74–14.52)	0.092
**Molecular subtype**								
Luminal A-like	–	–			–	–		
Luminal B-like	1.06 (0.43–2.64)	0.896			0.92 (0.22–3.85)	0.905	0.32 (0.06–1.68)	0.133
HER2 overexpression	0.67 (0.17–5.47)	0.709			NA	NA	NA	NA
TNBC	2.41 (0.76–7.72)	0.137			5.68 (1.33–24.28)	0.019	5.12 (1.04–15.15)	0.044
**ER status**								
Negative	–	–			–	–		
Positive	0.72 (0.29–1.79)	0.474			0.35 (0.12–1.08)	0.069		
**PR status**								
Negative	–	–			–	–		
Positive	0.47 (0.21–1.04)	0.064			0.37 (0.13–1.12)	0.078		
**HER2 status**								
Negative	–	–			–	–		
Positive	1.49 (0.59–3.72)	0.398			0.90 (0.20–4.08)	0.889		
**Lympho-vascular invasion**								
No	–	–			–	–		
Yes	1.28 (0.59–2.76)	0.532			1.52 (0.51–4.56)	0.453		
**Radiation therapy**								
No	–	–	–	–	–	–		
Yes	0.32 (0.13–0.74)	0.008	0.28 (0.12–0.67)	0.004	0.17 (0.04–0.77)	0.021	0.09 (0.02–0.44)	0.003
**Chemotherapy**								
No	–	–			–	–		
Yes	0.82 (0.33–2.03)	0.812			1.26 (0.28–5.70)	0.763		
**Endocrine therapy**								
No	–	–	–	–	–	–		
Yes	0.42 (0.20–0.93)	0.029	0.38 (0.17–0.84)	0.016	0.42 (0.14–1.27)	0.122		
**Targeted therapy**								
No	–	–			–	–		
Yes	1.01 (0.35–2.93)	0.880			0.48 (0.06–3.68)	0.477		

*ER* estrogen receptor, *PR* progesterone receptor, *HER2* human epidermal growth factor receptor 2, *ENE* extranodal extension, *SLN* sentinel lymph node, *TNBC* triple negative breast cancer.

**Figure 6:**
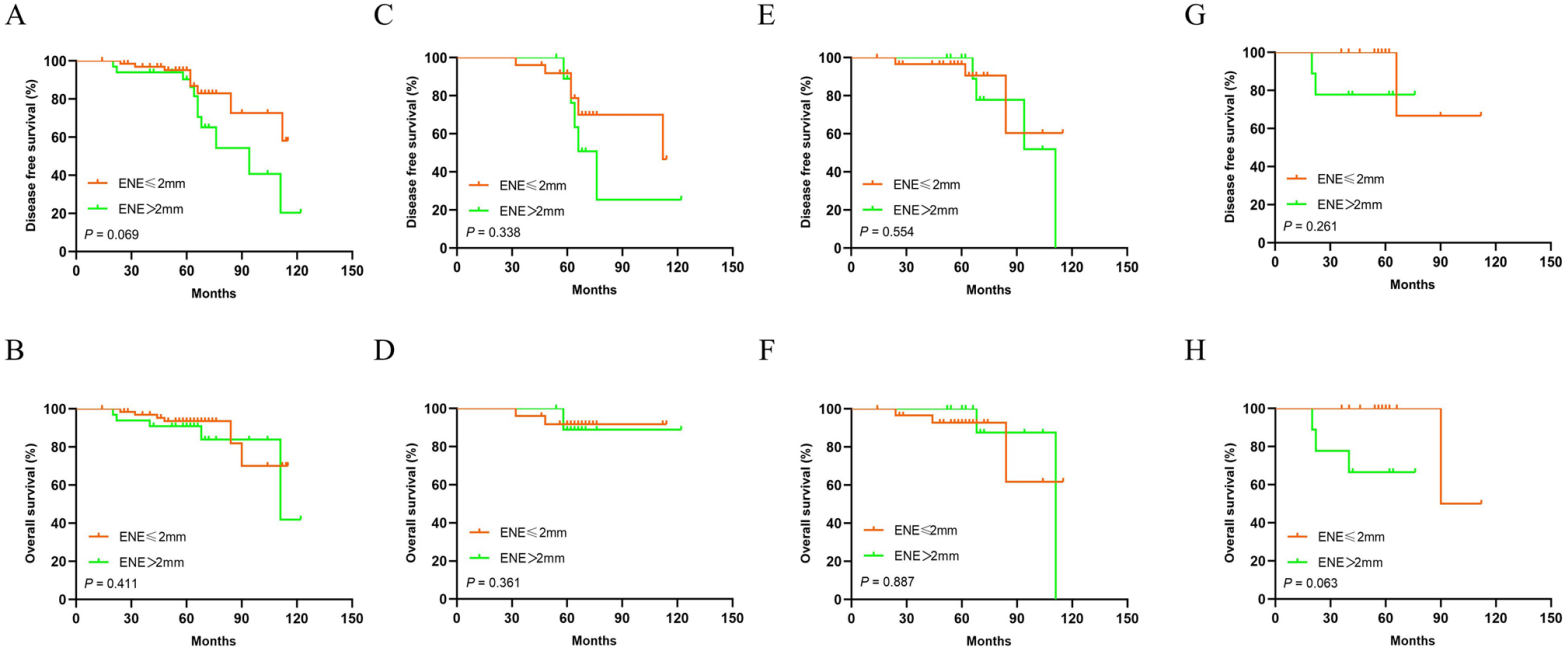
Kaplan–Meier curves depicting associations of ENE extent in SLN with DFS and OS in SLN positive patients with different nodal (N) stage. Comparison of survival rate for DFS (ENE ≤ 2 mm group vs. ENE > 2 mm group: *P* = 0.069) (**A**) and OS (ENE ≤ 2 mm group vs. ENE > 2 mm group: *P* = 0.411) (**B**) in patients with different size of ENE in SLN. Comparison of survival rate for DFS (ENE ≤ 2 mm group vs. ENE > 2 mm group: *P* = 0.338) (**C**) and OS (ENE ≤ 2 mm group vs. ENE > 2 mm group: *P* = 0.361) (**D**) between different ENE groups in patients with pN1 stage. Comparison of survival rate for DFS (ENE ≤ 2 mm group vs. ENE > 2 mm group: *P* = 0.554) (**E**) and OS (ENE ≤ 2 mm group vs. ENE > 2 mm group: *P* = 0.887) (**F**) between different ENE groups in patients with pN2 stage. Comparison of survival rate for DFS (ENE ≤ 2 mm group vs. ENE > 2 mm group: *P* = 0.261) (**G**) and OS (ENE ≤ 2 mm group vs. ENE > 2 mm group: *P* = 0.063) (H) between different ENE groups in patients with pN3 stage.

Moreover, survival analysis showed that patients with ENE > 2 mm had similar DFS and OS comparing with those with ENE ≤ 2 mm (for DFS: *P* = 0.069; and for OS: *P* = 0.411 respectively) (Fig. 6A-B). Patients with ENE larger than 2 mm had similar DFS and OS comparing with those with ENE no larger than 2 mm (for DFS: *P* = 0.338; and for OS: *P* = 0.361 respectively) in pN1 stage (Fig. 6C-D), (for DFS: *P* = 0.554; and for OS: *P* = 0.887 respectively) in pN2 stage (Fig. 6E-F), and (for DFS: *P* = 0.261; and for OS: *P* = 0.063 respectively) in pN3 stage (Fig. 6G-H). Cox proportional hazards regression analyses indicated that the size of ENE subdivided by a 2 mm cutoff value was not an independent factor for DFS or OS in 100 patients with ENE in SLN (Table 5).

**Table 5:** Correlations between the size of ENE subdivided by a 2 mm cutoff and prognosis (DFS and OS).

Variables	Disease free survival				Overall survival			
	Univariate analysis		Multivariate analysis		Univariate analysis		Multivariate analysis	
	HR (95% CI)	*P* value	HR (95% CI)	*P* value	HR (95% CI)	*P* value	HR (95% CI)	*P* value
**Age (Y)**								
< 50	–	–			–	–		
≥ 50	0.97 (0.39–2.38)	0.941			0.47 (0.13–1.79)	0.27		
**Tumor size**								
T1	–	–			–	–		
T2	3.56 (1.39–9.13)	0.008	2.38 (1.06–8.46)	0.048	5.33 (1.37–20.74)	0.016	1.85 (1.05–7.82)	0.018
**Histological grade**								
2	–	–			–	–		
3	4.08 (1.17–13.74)	0.027	2.99 (1.26–8.44)	0.039	6.34 (0.81–20.79)	0.079		
**N stage**								
1	–	–			–	–		
2	0.56 (0.22–1.46)	0.237			2.22 (0.43–11.58)	0.343		
3	0.46 (0.11–2.10)	0.316			4.46 (0.81–24.60)	0.086		
**ENE**								
≤ 2 mm	–	–			–	–		
> 2 mm	2.22 (0.10–5.39)	0.079	1.57 (0.25–3.95)	0.451	1.64 (0.50–5.38)	0.416	1.84 (0.23–5.68)	0.587
**Molecular subtype**								
Luminal A-like	–	–			–	–		
Luminal B-like	0.84 (0.29–3.43)	0.751			0.68 (0.16–2.90)	0.611		
HER2 overexpression	1.36 (0.17–7.91)	0.776			NA	NA		
TNBC	1.58 (036–5.56)	0.552			3.01 (0.55–13.49)	0.202		
**ER status**								
Negative	–	–			–	–		
Positive	0.68 (0.22–2.08)	0.501			0.46 (0.12–1.77)	0.261		
**PR status**								
Negative	–	–			–	–		
Positive	0.51 (0.19–1.38)	0.187			0.42 (0.12–1.47)	0.175		
**HER2 status**								
Negative	–	–			–	–		
Positive	2.45 (0.91–6.57)	0.075			1.33 (0.28–6.32)	0.721		
**Lympho-vascular invasion**								
No	–	–			–	–		
Yes	0.45 (0.19–1.09)	0.076			0.43 (0.13–1.42)	0.165		
**Radiation therapy**								
No	–	–			–	–		
Yes	0.24 (0.09–0.68)	0.006	0.24 (0.08–0.70)	0.008	0.08 (0.10–0.62)	0.015	0.08 (0.01–0.65)	0.018
**Chemotherapy**								
No	–	–			–	–		
Yes	0.54 (0.20–1.49)	0.232			0.68 (0.15–3.18)	0.628		
**Endocrine therapy**								
No	–	–			–	–		
Yes	0.57 (0.21–1.54)	0.271			0.68 (0.18–2.64)	0.58		
**Targeted therapy**								
No	–	–			–	–		
Yes	1.94 (0.64–5.90)	0.243			0.82 (0.10–5.50)	0.852		

Abbreviations: ER estrogen receptor; PR progesterone receptor; HER2 human epidermal growth factor receptor 2; ENE extranodal extension; SLN sentinel lymph node; TNBC triple negative breast cancer.

## Discussion

Invasive breast cancer is the most common malignancy in women. The most common metastasis site of breast cancer is axillary lymph nodes, and SLN is the first station of nodal metastasis^1,2^. Recently, a study about the relationship between Oncotype-DX recurrence score (RS) and lymph node burden in clinically node negative breast cancer patients found that RS couldn’t predict nodal burden and wasn’t useful to guide decisions regarding the extent of axillary surgery^28^. Therefore, it is necessary to find a useful histological marker that can identify those patients who have a high risk to Non-SLN nodal metastasis and poor prognosis.

ENE has been recognized as a prognostic predictor in several types of malignancies^16,29–32^, and has been included in AJCC TNM staging system of head and neck cancers^33,34^, which also has been required to be described in routine pathological reports according to the College of American Pathologists (CAP)^35^. However, it has not yet been included in the eighth edition of AJCC Cancer staging system of breast cancers^36^. Some studies have demonstrated that the presence of ENE in involved axillary nodes was associated with total number of involved axillary nodes and poor prognosis in breast cancer^37–39^. However, the predictive and prognostic significance of ENE in SLN still need further investigation.

In this retrospective analysis including 266 breast cancers with SLN involvement, we found 100 patients (37.6%) were ENE positive in SLN. The rate of ENE in SLN in this study was compatible with previous reports^25,40,41^. Among the clinicopathologic characteristics examined in the cohort, we found that ENE in SLN had a significantly association with higher pT and pN stage, PR status, lympho-vascular invasion. Meanwhile, an excellent interobserver agreement between two observers was demonstrated in ENE evaluation in our study. It may be feasible to evaluate ENE in SLN in routine practice.

Previous studies have demonstrated the presence of ENE in SLN was associated with overall nodal burden^21,22,42,43^. Some studies showed that ENE in SLN was associated with four or more metastatic axillary nodes^20,25,44^. ENE in the involved lymph node was regarded as a demonstration of tumor migration and invasion ability which recruit degradation factors that permit cancer cells to break through the lymph node capsule^22,41,45^. In our cohort, patients with ENE in SLN had significantly higher frequency of non-SLN involvement and higher nodal burden. The presence of ENE in SLN was significantly positively correlated with non-SLN metastasis in univariate and multivariate analysis. We also built a nomogram including ENE to predict non-SLN metastasis for an individual patient. Higher frequency of pN2 disease, higher number of involved SLN, non-SLN metastasis and total positive LNs was observed in ENE positive group. Whether additional axillary node dissection is necessary in patients with SLN micrometastasis or only 1–2 SLNs involvement is still controversial. In our study, in patients with SLN micrometastasis or 1–2 SLNs involvement, ENE positive patients had higher rate of non-SLN metastasis, comparing with ENE negative patients. Our study indicated that ENE in SLN was a significant predictor for non-SLN involvement and nodal burden, and such patients may benefit from additional ALND, even in SLN micrometastasis or 1–2 SLNs involvement patients.

The prognosis value of ENE in SLN is still in exploration. A meta-analysis including 624 patients (163 ENE + and 461 ENE-) showed that ENE in SLN was associated with a higher risk of both mortality (RR = 2.51; 95% CI 1.66–3.79, *P* < 0.0001) and recurrence of disease (RR = 2.07, 95% CI 1.38–3.10, *P* < 0.0001)^46^. Schwentner L et al. found that ENE in SLN was linked to worse overall survival in univariate analysis, while this correlation disappeared when adjusting for nodal status, age, and comorbidities in multivariate analysis^45^. Similar results have been found in study that conducted by Choi et al.^25^. Other studies which had relatively small population indicated that the presence of ENE in SLN was associated with poorer prognosis^41,47^. In our study, Kaplan Meier curves and log-rank test showed that ENE in SLN was associated with lower DFS and OS. Cox proportional hazards regression analyses showed that the presence of ENE in SLN was an independent predictive marker for DFS both in univariate and multivariate analysis. ENE was associated with OS in univariable analysis but not in multivariable analysis. According to these findings, ENE in SLN had significant predictive values for prognosis in breast cancers.

Methodologies for ENE size measurement is still not standardized^41,45,47^. Aziz et al. evaluated the clinical significance of ENE which was divided into circumferential (CD-ENE) and perpendicular (PD-ENE) extra-nodal growth, and the results showed that PD-ENE (with 3 mm as cut-off value) was an independent prognostic factor for disease-free survival of breast cancers^48^. Choi et al. and Gooch et al.’s study showed that the extent of ENE was associated with greater axillary disease burden, and ENE > 2 mm was the strongest predictor of N2 disease (*P* < 0.001), and poorer DRFS and OS^25,40^. The 2017 AJCC TNM classification of head and neck cancer^33^ classified ENE into ENEmi and ENEma based on a 2-mm cutoff value for the extension distance of cancer cells from the lymph node capsule. In our study, we measured the extent of ENE by the highest or widest diameter of the invasive front and set a 2 mm cutoff value. 67 (25.2%) had ENE ≤ 2 mm, and 33 (12.4%) had ENE > 2 mm. Our study showed that there was no significant difference in nodal burden between these two groups, except higher number of involved SLN in ENE > 2 mm group. Cox proportional hazards regression analyses indicated that the size of ENE was not an independent factor for DFS and OS in patients with ENE in SLN, which indicated that the extent of ENE in SLN that subdivided by a 2 mm cutoff value had no significant prognosis value in breast cancer, and the cutoff values of ENE in SLN still need further exploration.

Meanwhile, our study had some limitations. It was a single-institution retrospective analysis including relatively small samples. Further large-scale prospective and retrospective studies still need to evaluate the clinical values of ENE in breast cancer. The cutoff values of ENE in SLN still need further investigation.

### Ethics approval and consent to participate

The study was approved by Ethics Institutional Review Board of Fudan University Shanghai Cancer Center. All procedures performed involving human participants were in accordance with the ethical standards of Ethics Institutional Review Board of Fudan University Shanghai Cancer Center and with the 1964 Helsinki declaration and its later amendments. Written informed consent was obtained from all patients of the study, who signed the informed consent allowing the use of their biological material, donated for our Biobank, for scientific projects, and for data publication.

## Conclusions

Our study indicated that ENE in SLN was a predictor for non-SLN metastasis, nodal burden and prognosis in breast cancers. Patients with ENE in SLN had higher rate of non-SLN metastasis, higher nodal burden, higher frequency of pN2 disease, and poorer prognosis. Patients with ENE in SLN may benefit from additional ALND, even in SLN micrometastasis or 1–2 SLNs involvement patients. The presence of ENE in SLN should be evaluated in clinical practice. The size of ENE which was classified by a 2 mm cutoff value had no significant predictive and prognostic values in this study. The cutoff values of ENE in SLN need further investigation.
